# Beyond limits: a tribute to Dai-Sik Kim’s academic legacy and vision

**DOI:** 10.1515/nanoph-2025-0394

**Published:** 2025-10-16

**Authors:** Jae Sung Ahn, Kwang Jun Ahn, Young-Mi Bahk, Geunchang Choi, Jeeyoon Jeong, Young-Gyun Jeong, Taehee Kang, Hyun Woo Kim, Richard H.J. Kim, Sunghwan Kim, Dukhyung Lee, Geon Lee, Joong Wook Lee, Kwang-Geol Lee, Woongkyu Park

**Affiliations:** Smart Gym-Based Translational Research Center for Active Senior's Healthcare, Pukyong National University, Busan 48513, Republic of Korea; Department of Energy Systems Research, Ajou University, Suwon 16499, Republic of Korea; Department of Physics, Incheon National University, Incheon 22012, Republic of Korea; School of Electrical and Electronics Engineering, Chung-Ang University, Seoul 06974, Republic of Korea; Department of Semiconductor Physics and Institute of Quantum Convergence Technology, Kangwon National University, Chuncheon 24341, Republic of Korea; The Edward S. Rogers Sr. Department of Electrical and Computer Engineering, University of Toronto, Toronto, ON M5S 3G4, Canada; Sensor System Research Center, Korea Institute of Science and Technology (KIST), Seoul 02792, Republic of Korea; Drug Discovery Research Center, Korea Research Institute of Chemical Technology (KRICT), Daejeon 34114, Republic of Korea; Ames National Laboratory, US Department of Energy, Ames, IA 50011, USA; Center for Multidimensional Carbon Materials (CMCM), Institute for Basic Science (IBS), Ulsan 44919, Republic of Korea; School of Applied and Engineering Physics, Mohammed VI Polytechnic University, Ben Guerir 43150, Morocco; Department of Physics and Optoelectronics Convergence Research Center, Chonnam National University, Gwangju 61186, Republic of Korea; Department of Physics, Hanyang University, Seoul 04763, Republic of Korea; Photonics Energy Components Research Center, Korea Photonics Technology Institute (KOPTI), Gwangju 61007, Republic of Korea

**Keywords:** subwavelength optics, near-field optics, terahertz plasmonics

## Abstract

This article is dedicated to the cherished memory of Prof. Dai-Sik Kim, a visionary leader and an inspiring mentor who profoundly shaped international as well as domestic landscape of optical science, and addresses topics that were central to his scientific passion in nanophotonics. Prof. Kim’s research spanned surface plasmonics, near-field optics, and terahertz plasmonics and spectroscopy, each reflecting his enduring commitment to exploring the interaction between light and matter beyond conventional limits.

## 1 Introduction

**Kwang Jun Ahn** *Ajou University*

Dr. Dai-Sik Kim born in 1963 in Seoul, South Korea, was a distinguished physicist whose innovative research reshaped key areas of nanophotonics and terahertz (THz) optics. He earned his B.S. in Physics from Seoul National University (SNU) in 1985, followed by an M.A. in Biophysics (1986) and a Ph.D. in Physics (1990) from the University of California, Berkeley. He conducted postdoctoral research at Oklahoma State University (1990–1991 and 1993–1994) and AT&T Bell Laboratories (1991–1993). In 1994, he returned to South Korea and joined the Department of Physics at SNU where he would later be recognized as a central figure in Korean optics research. In 2019, he accepted a position at Ulsan National Institute of Science and Technology (UNIST) as a Distinguished Professor of Nano Optics. He also served as Director of the Quantum Photonics Institute at UNIST. In 2023, he was appointed Emeritus Professor at SNU.

Prior to 2002, Dr. Dai-Sik Kim’s scientific research concentrated on unraveling ultrafast phenomena in condensed matter systems, primarily utilizing the powerful four-wave mixing (FWM) spectroscopy technique. His work in this period was instrumental in understanding the fundamental dynamics of photo-excited states, coherent interactions, and dephasing processes in various materials, particularly in semiconductors and strongly correlated electron systems [1], [2], [3], [4]. Through FWM, he unraveled the intricate interplay between light and matter on femtosecond timescales, providing critical insights into quantum mechanical processes. These investigations, marked by a deep curiosity for the fundamental, laid the groundwork for his remarkable contributions in nanophotonics.

From 2002 onward, Dr. Kim’s research broadened to include plasmonics, metamaterials, and light–matter interactions at atomic scales in a broad spectral range from THz to visible light. His pioneering work in plasmonics, particularly in the development of plasmon-enhanced near-field optical microscopy, subwavelength optics, THz quantum plasmonics, active plasmonic devices, significantly advanced this field. His work not only pushed the boundaries of fundamental physics but also paved the way for practical devices with enhanced performance and novel functionalities. These efforts significantly inspired researchers worldwide to pursue ambitious goals in this exciting field. In recognition of his scientific excellence, he received the Korea Young Scientist Award in 2002, the Korea Science Award in 2013, and the Humboldt Research Award in 2024. He was also elected a Fellow of Optica (formerly OSA) and the American Physical Society (APS) in 2010.

Beyond his core expertise in physics and nanophotonics, Dr. Kim also demonstrated an unconventional and broad scientific curiosity, venturing into areas seemingly disparate from his major field. Notably, he co-authored several medical articles on male circumcision, examining its prevalence, historical context, societal factors, and effects on sexuality in middle-aged South Korean males. These unique contributions, published in medical and public health journals [5], [6], showcased his willingness to apply rigorous scientific inquiry to complex social and health phenomena, reflecting a truly interdisciplinary and inquisitive mind. His advocacy earned international recognition, including a Human Rights Award from the International Symposium on Genital Integrity in 2000.

The loss of Prof. Kim in 2024 reverberated throughout the global optics and photonics community. He was not just a leading academic, but was a mentor, a motivator, and a guiding light for countless students and colleagues. His influence lives on, not only in the groundbreaking studies he authored but also in the vibrant scientific community he cultivated. That community continues to explore the frontiers of quantum and nanoscopic light–matter interaction guided by the trail he blazed.

**Figure:**
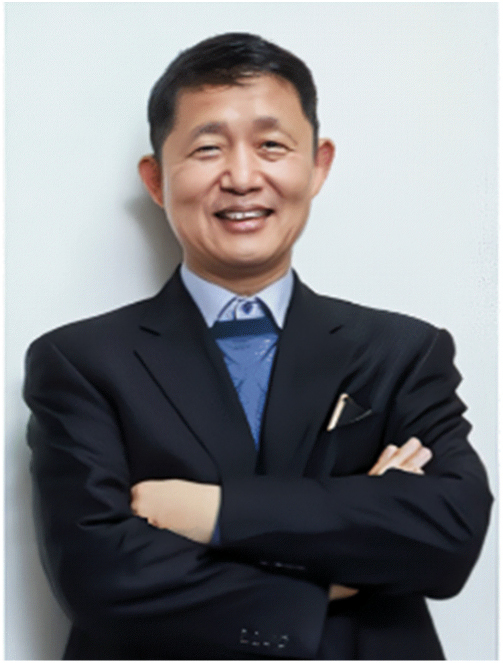


## 2 Near-field and subwavelength optics

**Kwang-Geol Lee** *Hanyang University*

**Hyun Woo Kim** *Korea Research Institute of Chemical Technology (KRICT)*

Through his endless stream of ideas and questions, Dai-Sik would draw his students into the stage of research unfolding in his mind, and we found ourselves immersed with him in a boundless sea of curiosity. This is a short story of the wide-ranging legacy he left in the study of optics within the tiny realm of the nanoscale.

Professor Kim’s journey into nano-optics began somewhat unexpectedly. Until then, his group had been actively engaged in studying semiconductor properties and carrier dynamics using femtosecond pump-probe spectroscopy. The spark of curiosity for nano-optics was first ignited by Prof. Sungchul Hohng (now at SNU, South Korea), who, at the time as a graduate student, introduced Thomas Ebbesen’s work on extraordinary optical transmission (EOT) through plasmonic nanohole arrays [7]. At first, Prof. Kim responded with mild interest, but soon after, with his characteristic declaration, “Let’s do NANO too,” our lab’s exploration of nano-optics was underway. Key collaborators included Prof. Christoph Lienau’s group at the Max-Born Institute (MBI) in Germany and Prof. Q-Han Park’s team at Korea University in South Korea.

In the early studies, the mechanism by which surface plasmon polariton (SPP) components contribute to the far-field was investigated through near- to far-field measurements of plasmonic hole arrays [8]. This was followed by the identification of SPP damping mechanisms via Rayleigh scattering, and quantitative characterization of the lineshape through measurements of the coherent propagation length and decay lifetime [9]. This work was carried out during his visit to the MBI, and I still remember how delighted he looked as he analyzed the experimental data he had obtained himself. Subsequent research demonstrated subradiant damping of SPPs in antisymmetric modes [10], and introduced the dressed SPP (DSPP) model to explain the detailed features of far-field transmission spectra [11].

**Figure 1:**
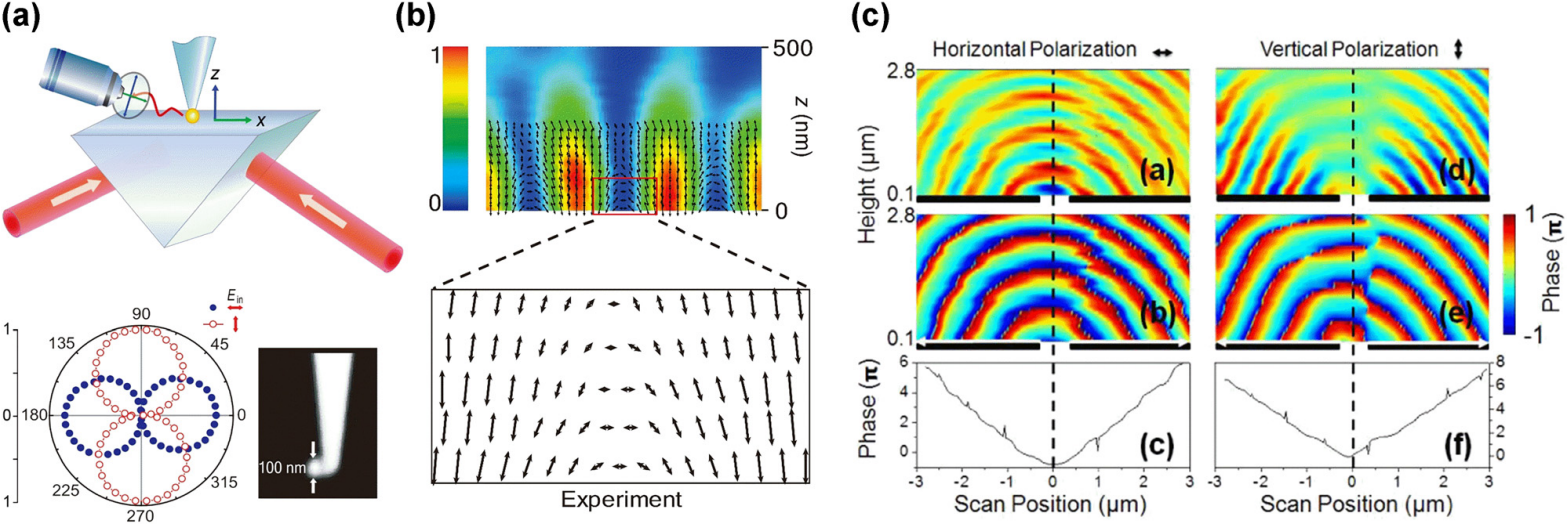
Vector-field microscope for imaging of SP and surface diffracted light. (a) (Top) Schematic of 1st generation vector-field microscopy. Far-field scattered light is polarization analyzed to reconstruct the local field direction and magnitude. (Down) Tip characterization: polarization-resolved scattering response of a gold nanoparticle-attached tip (SEM image) for horizontal and vertical electric fields. Reproduced from Ref. [12]. (b) Vector-field image of a standing SPP wave on a gold surface. Reproduced from Ref. [12]. (c) Near-field images of SP and surface-diffracted modes emanating from a nanoslit in gold film, obtained by 2nd generation vector-field microscope combined with optical interferometer. Reproduced from Ref. [17].

These early findings provided deeper insights into the underlying mechanisms of SPPs in nanostructures, marking a pioneering contribution to the advancement of plasmonics. Furthermore, the initial near-field investigations laid the groundwork for a new range of nano-optics research directions, including vector-field microscopy, magnetic field imaging, and the funneling of electromagnetic waves across visible to THz frequencies.

### 2.1 Vector-field microscopic imaging

**Kwang-Geol Lee** *Hanyang University*

In the early stages of efforts to understand EOT, interpretations regarding the respective roles of SPPs and diffracted surface optical waves were varied and often contentious. To deepen our understanding of the mechanisms through which SPPs operate – particularly in distinguishing their contributions from other surface modes – it became necessary to separately measure the horizontal and vertical components of the near-field electric field. This need led to the collaborative development of a novel methodology with our research partners.

Through detailed analysis of scattering signals from a nanoscopic probe, we established an imaging technique that visualizes electric lines of force, a method later termed vector-field microscopy [12]. Although the concept was unfamiliar and initially met with some skepticism [13], it ultimately established the foundation for advanced vector-field imaging techniques. With a quantitative and comprehensive characterization of the nano-probe [14], the technique evolved from two-dimensional imaging to full three-dimensional mapping [15]. This technique was utilized for the study of image dipole effect [16]. Combined with optical interferometry, vector-field microscope had been further developed to enable near-field imaging of propagating wavefronts of SP and optical diffraction modes emanating from nanoslits, which provided crucial information to discriminate between SP and diffraction modes based on different optical phases depending on their electric and magnetic field components [17]. Today, vector-field microscopy is widely used to experimentally investigate light–matter interactions in nanostructures with high precision and has become an essential tool in the analysis and design of plasmonic structures, photonic crystals, and metamaterials (Figure 1).

### 2.2 Subwavelength aperture optics – optical magnetism

**Hyun Woo Kim** *KRICT*

Optical-frequency electromagnetic waves consist of electric and magnetic fields oscillating at frequencies of 10^15^ Hz. The degree to which these electric and magnetic fields interact with matter is described by permittivity (*ε*) and permeability (*μ*). Unlike in the low-frequency domain where magnetic fields significantly influence materials, the magnetic field barely interacts with matter in optical frequencies with *μ* approaching a value of 1. The stark contrast in the strength of photon-matter interaction in electric and magnetic fields is due to the difference in the speed with which electrons respond to electric and magnetic fields. Electrons can respond almost instantaneously to the rapid oscillations of electric fields, while their response to magnetic fields is much slower because electrons should have a closed-loop current path to respond to a magnetic field.

Limitation on optical magnetic field to interact with matter can be loosened in artificially engineered metamaterials, where closed-loop patterned nanostructures often show non-unity *μ* values. Dai-Sik focused on the crucial role of magnetic fields in near-field optical phenomena and studied their behavior in one-dimensional slit and circular hole structures. In the non-optical wavelength regime (THz and microwaves), where metals behave nearly as perfect conductors, he experimentally revealed the crucial role of magnetic fields in extraordinary transmission through slit structures [18]. However, finding how magnetic fields affect optical phenomena was not trivial because the assumption of perfect conductivity breaks down in the optical regime due to significant damping in metals. The most important challenge was rather philosophical- can really we observe optical magnetic field even though we are having only electric field-sensitive detectors (photodiode, fluorescence dye)? Nanoparticles with magnetic dipoles allowed for transition, and metamaterials with magnetic modes could be good detectors for optical magnetism. However, Dai-Sik was obsessed to facilitate nanostructures with more simple design to hunt optical magnetic fields.

**Figure 2:**
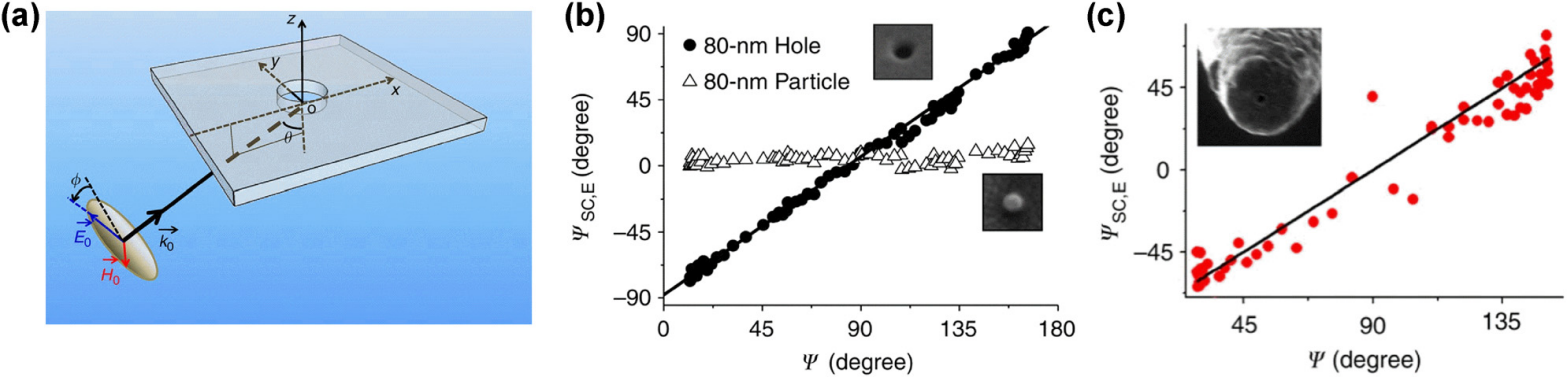
Optical magnetism in metallic apertures. (a) Schematic of the experimental setup for polarization analysis of incident and transmitted light through a metallic aperture. Reproduced from Ref. [19]. (b) Polarization change of the transmitted light through the aperture (filled circles) and of the scattered light from a nanoparticle (open triangles), both as a function of the incident polarization. Reproduced from Ref. [19]. (c) Polarization-dependent transmission through a metallic aperture located at the apex of a near-field scanning optical microscope (NSOM) probe. Reproduced from Ref. [19].

Dai-Sik has achieved the complex task with careful polarization analysis of the incident-transmitted-scattered light from metallic nano apertures. Circular metallic apertures are formed at the apex of near-field probes or in thin metal films, and the transmitted light through the apertures was analyzed in accordance with incident polarization change clearly demonstrating the role of the magnetic dipole formed in the aperture [19]. Compared to optical magnetic probe demonstrated by Kuipers group in the Netherlands [20], Dai-Sik’s work is notable for demonstrating that the theoretical magnetic dipoles proposed by Hans Bethe [21] – originally applied to the radio frequency domain – also exist in the optical regime. This finding is significant because it provides a foundational structure that can be used to enhance optical magnetic field interactions in the design of complex metamaterials. Dai-Sik always enjoyed to be with his students and having intense discussions on their experimental results. He put logical clarity and quality of data on first priority in doing science in entire his research career. He was rather night owl type person who often bulge in lab in middle of night without any notice to have discussion on his idea. I would never be able to forget his glaring eye inquiring the interpretation of my data and next plan (Figure 2).

### 2.3 Subwavelength aperture optics – funneling of electromagnetic waves

**Jae Sung Ahn** *Pukyong National University*

Prof. Dai-Sik Kim was a transformative figure in the field of nanophotonics, profoundly advancing the understanding of confinement and enhancement of electromagnetic waves within nanometer-sized metallic gaps across a broad frequency spectrum, from optical to microwave ranges. His pioneering research not only pushed theoretical boundaries but also established robust experimental methodologies to quantify these phenomena. In describing the funneling electromagnetic waves through subwavelength apertures, Prof. Kim would often quote Scripture, referencing Matthew 19:24: “It is easier for a camel to go through the eye of a needle than for someone who is rich to enter the kingdom of God.”

#### 2.3.1 Optical and near-infrared (NIR) regimes

In the optical to near-infrared range (0.6–2.3 µm), Prof. Kim advanced the experimental quantification of electric field enhancement in nanogaps by employing both far-field interferometry and NSOM. Through the application of Kirchhoff integral formalism to far-field transmission measurements [22], [23], and direct optical probing via NSOM [24], he independently verified consistent field enhancement factors across both methods. The optical field enhancement in nanogaps could not be explained by simple capacitive charging models and was instead attributed to strong gap plasmon resonance – exhibiting Fabry–Pérot-like behavior tunable by gap geometry. While field enhancement in the THz regime typically follows a non-resonant 1/*f* decay, the near-infrared regime revealed sharp resonance peaks that elevated field intensities well beyond classical predictions. These findings established that gap plasmon resonance, rather than capacitive effects, plays a dominant role in governing field enhancement at optical frequencies.

#### 2.3.2 THz regime

Prof. Kim made groundbreaking contributions in the THz regime (0.1–1.1 THz), demonstrating field enhancement in metallic nanoslits beyond the skin-depth limit [25]. These slits effectively function as nano-gap capacitors charged by light-induced currents, leading to exceptional field enhancement. For instance, a 70 nm gap showed an electric field enhancement of up to 800 times at 0.1 THz, a phenomenon occurring at a frequency where gold’s skin depth is 250 nm. This remarkable finding indicated that the field is highly concentrated within the nanogap without significant penetration into the metal. The observed enhancement displayed a resonance-lacking 1/*f*-type frequency dependence, consistent with a capacitor-like charging mechanism.

#### 2.3.3 Microwave regime

Prof. Kim’s research further extended the limits of light confinement into the microwave regime, achieving funneling through sub-10 nm metallic nanogaps at a remarkable *λ*/10,000,000 scale [26]. By fabricating structures with extreme aspect ratios, such as 300-nm-wide, 3.5-mm-long nano-slots and sub-10-nm-wide rectangular nanogap rings with a perimeter of 6.5 mm, he reported substantial microwave transmittance (up to 50 %). Given the minute area covered by these gaps, this implies an electric field enhancement within the nanogaps of up to 1,400 times for nano-slots and up to 5,000 times for nanogap rings, with an estimated intensity enhancement factor reaching up to 25 million. This effective funneling mechanism is attributed to the capacitive coupling of induced charges at the sidewalls of the metallic gap, further verified by a high polarization extinction ratio of up to 20 dB. This breakthrough demonstration of resonant microwave transmission through nanometer-sized structures opens new avenues for low-frequency applications, including centimeter-wave nonlinearities and enhanced detection sensitivities.

## 3 THz plasmonics

**Joong Wook Lee** *Chonnam National University*

While the newly emerging field of plasmonics has been widely explored in the visible and infrared ranges, the extension to the THz regime opens new opportunities for manipulating the THz waves at subwavelength scales and for implementing novel THz devices. Early research primarily focused on demonstrating extraordinary transmission in the THz regime through plasmonic materials with periodic structures based on a variety of shapes, including holes and slots, and on gaining a fundamental understanding of the mechanisms behind this phenomenon [27], [28], [29], [30], [31]. Such plasmonic metamaterials exhibit perfect transmission at specific resonance frequencies primarily determined by their geometric shape and periodicity, which has been exploited for realizing versatile THz filters. In the case of one-dimensional thick metallic structures, the phenomenon has been explained in relation to Fabry–Pérot resonance occurring within the spectral wavelength region below the Rayleigh minimum. Otherwise, for thin metallic structures, it has been observed that the THz transparency is governed by the fundamental shape resonance of individual unit cells, such as rectangular holes and slits that constitute the plasmonic metamaterials. Of particular interest to us was that the extremely high transmittance in the plasmonic metamaterials is associated with the strong concentration of near-electric fields within individual unit cells [32]. This implies that properly scaling specific dimensions of the unit structure can lead to enormous field enhancement perceived as a result of the electromagnetic funneling effect. This concept enabled the beginning of a long journey from micron-gaps to nanogaps, and eventually to angstrom-scale gaps.

### 3.1 THz subwavelength optics (from micron to angstrom scale)

**Sunghwan Kim** *Ulsan National Institute of Science and Technology (UNIST)*

**Young-Mi Bahk** *Incheon National University*

Prof. Dai-Sik Kim’s fundamental and insightful contributions to the understanding of the interaction between diverse metal holes and THz waves inspired us to explore THz subwavelength optics, particularly in relation to metal gap sizes. Early on, he demonstrated perfect transmission at specific frequencies using various slots and slits with micron-scale widths, introducing the concept of funneling phenomena of electromagnetic waves and electric field enhancement in the metal gap within the THz range [32]. It was subsequently discovered that the electric field enhancement strongly depends on the gap size [33]. Remarkably, even when the gap size was reduced to hundreds or tens of nanometers – much smaller than the THz wavelength (with the ratio of wavelength to gap size *λ*/w being approximately 10^4^) – and when the metal thickness is below the skin-depth of the metal, THz waves were still able to effectively funnel into the nanogap, yielding field enhancement exceeding 1,000 [25].

Building on this, we successfully developed a fabrication method that combined conventional UV lithography with atomic layer deposition (ALD), enabling the creation of millimeter-long, few-nanometer-wide metal gaps [34]. This advancement led to a field enhancement of 10,000, allowing us to explore the saturation of electric field enhancement in slit structures [35]. Our understanding of the funneling phenomenon – across the micron to nano-scale metal gaps – emerged from a detailed analysis of the distinct physical mechanisms at play in different gap size regimes [36]. In particular, the saturation of electric field enhancement was subsequently understood as a classical limit, governed by the wavelength and metal film thickness, before entering the quantum regime.

Ultimately, our research reached the quantum realm through an angstrom-scale metal gap (*λ*/*w* ∼ 10^7^) using atomically thin graphene sheets as spacers between two metal films [37]. At this scale, nonlinear THz transmission, attributed to quantum tunneling effects, was observed, resulting from the extremely high electric fields in the ultra-narrow gaps. Furthermore, we demonstrated that the width of such gaps, created from multi-layer graphene, could be precisely tuned at the angstrom scale by adjusting the number of graphene layers – each corresponding to the van der Waals distance between layers [38].

**Figure 3:**
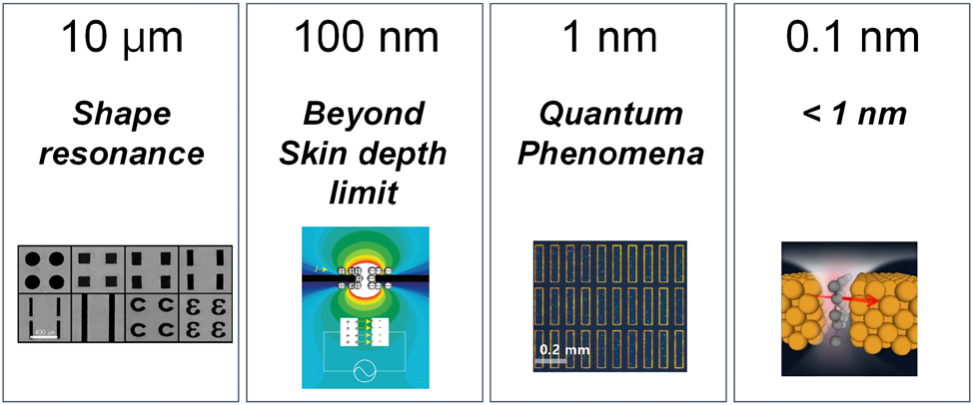
Overview of Prof. Dai-Sik Kim’s exploration of THz subwavelength optics. His guidance, through the fundamental and insightful questions, has advanced the understanding of metallic structures interacting with THz waves by progressively reducing the gap size from the microscale to the angstrom scale. The key question at each scale is represented by a single crucial word. Images are adapted from Refs. [25], [29], [34].

Our understanding of the funneling phenomenon of electromagnetic waves has been further expanded in terms of geometric configuration of metal gaps and environmental constituents. Beyond the aforementioned field enhancement in metal slit structures, the transmission resonance of metal slot structures has been utilized to further understand the coupling effects between slot antennas, as well as to provide a platform for detecting adjacent systems. For instance, we explored how the resonant frequency and bandwidth undergo significant changes when different slots are placed very close to each other, at a scale much smaller than the wavelength or the metal’s skin depth [39], [40], [41], [42]. Furthermore, considering that field confinement extends beyond the gap into the fringe field region, we examined how the THz transmission characteristics of metal gaps are affected by the surrounding environment, such as thin substrates [43]. This deeper understanding of the arrangement of nanoslot antennas and the surrounding gap environment effects on resonance characteristics provides a foundation for exploring light–matter interactions between THz waves and nanomaterials, such as nanoparticles, nanofilms and nanosurfaces, through the use of THz nanoslot antennas (Figure 3).

### 3.2 THz quantum plasmonics

**Richard H. J. Kim** *Ames National Laboratory*

**Taehee Kang** *Korea Institute of Science and Technology (KIST)*

**Woongkyu Park** *Korea Photonics Technology Institute (KOPTI)*

With the advent of metallic nanoslits defined by gap sizes down to the ultimate single-atom thickness scale, it naturally became inevitable to encounter quantum effects in the new nano-THz platform. As THz electromagnetic waves squeeze through an angstrom-scale van der Waals gap, the effective polarization property of the gap media becomes strongly modified by electron tunneling driven by the THz electric field itself [37]. Thus, THz quantum plasmonics was born. Our contribution as graduate students in Professor Dai-Sik Kim’s group at that time was then to find out whether the quantum phenomena could be exploited in gaps of larger scale over 1 nm; a completely different environment of extremely low tunneling. Thanks to the strong field enhancements inside subwavelength nanogaps, we indeed observed a remarkable decrease in THz transmission from THz-funneling-assisted electron tunneling across the gap widths even up to 10 nm [44], [45]. The study newly extended quantum plasmonics to gap widths well over 1 nm by taking advantage of the low-energy THz frequency range. As electric fields are completely concentrated at the dielectric gap without penetration into the metal due to the high contrast of optical constant at this frequency, nonlinear transmission experiments can be conducted straightforwardly with signals explicitly originating from the gap [46]. Next, another big breakthrough came with the accomplishment of experimental quantification of the rectified tunneling currents in the THz nanogaps that possess macroscopic asymmetry [47], [48]. In this work, we introduced a new ultrafast optoelectronics platform that can control the direction of light-induced tunneling electrons by the global geometry and the local operation of optical sampling pulses coinciding with the THz-field illumination. This concept leads to unforeseen phenomena of ultrafast full-wave rectification of THz waves.

Another striking manifestation of tunneling electrons came in the form of a chemical reaction driven by THz electric fields. Whereas much of our earlier work focused on the electromagnetic behavior of ultranarrow gaps – including transmission suppression and rectified currents – Prof. Kim also explored how these extreme field conditions could directly trigger physical or chemical transformations within matter. In particular, he was interested in whether the tunneling electrons generated by THz fields in nanogaps could serve not only as passive indicators of quantum phenomena but also as active agents that initiate reactions. To address this, precursor studies were conducted on how strong localized fields confined in nanogaps could be used to drive resist material transformations. We developed a fabrication method combining photolithography, sacrificial overhangs, and ALD to produce sub-10 nm chromium nanogaps across centimeter-scale areas. These nanostructures functioned as contact-mode photomasks in the ultraviolet regime, leveraging evanescent field transmission through the gap to induce photoresist cross-linking – without relying on nonlinear optics or scanning probes [49]. After confirming the possibility of using evanescent-field lithography in the UV regime, we extended this concept to the THz regime, leveraging its strong electric fields, investigating whether intense THz fields could trigger localized chemical transformations. Using nanogap-based metallic antennas illuminated by broadband THz pulses, we demonstrated that the strongly enhanced electric fields within the gaps produced tunneling electrons capable of initiating polymerization of resist materials. Generally, the electron-resist is activated by external electron sources that trigger cross-linking between resist molecules. In contrast, this nanoscale electron-resist polymerization – realized within our THz plasmonic platform – was driven solely by THz-field-induced tunneling currents, without external beam irradiation [50]. This breakthrough opened the door to an advanced lithographic technique for fabricating ultranarrow features that persist across centimeter-scale areas.

**Figure 4:**
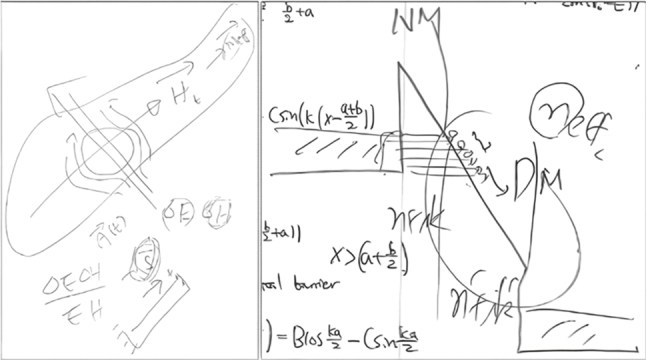
Dai-Sik Kim’s sketch on experimentally studying the Heisenberg’s uncertainty relation for electromagnetic waves (left) and his drawing and my (Richard Kim) overlayed notes of the tunneling barrier for THz quantum plasmonics (right).

These results all owe a large part to the peculiar footprint of the nanogap structure: the width being small as to allow only a handful of atoms to fit across the gap but the length along the slit reaching up to macroscopic dimensions of millimeters or even centimeters. We believe that THz quantum plasmonics is just the beginning of what Dai-Sik envisioned for these innovative nanostructures which seamlessly lead THz and millimeter wave technologies to meet with quantum mechanical effects. The idea of producing macroscopic THz coherence derived from nanoscale phenomena inherently relates nicely with the manifestation of quantum physics at a macroscopic level in today’s quantum materials involving electronic correlation and topology. Going back to electron tunneling, our THz slit experiment to study the change in the gap dielectric provides an intuitive picture on the interaction between light waves and electron matter waves in the same spirit as how looking through graphene lets one assess the fine structure constant by the naked eye [51]. Yet a deeper understanding would be required to elucidate fundamental questions, such as defining the tunneling time in the light-field-driven processes and the microscopic theory of describing the polarizability or the dielectric function in the presence of evanescent quantum states that are exponentially decaying at potential barriers. Also, intense electric pulses of light can pull down barriers of nanogaps in the fashion of tunnel ionization [52], potentially offering an on-chip broadband emitter based on high harmonic generation. I (Richard Kim) still remember my first one-on-one conversation with Dai-Sik in 2011, which was interestingly about quantum mechanics. It started with an unexpected overnight trip for me to Busan from Seoul to meet him at six in the morning in the lobby of Lotte Hotel just to get interviewed as a PhD student to join his group. He was chairing the SPP5 event held in Busan at the time. We moved to a dining table and talked about Heisenberg and wrote about his lesser-known uncertainty relation for electromagnetic fields nostalgically on a breakfast napkin which was used to test me if I qualified as an acceptable student. The episode perfectly showed his unorthodox character of urgency, dedication, and ambitiousness to take new territory. Through his pioneering research and his viewpoints on future opportunities, we will always remember Dai-Sik as our teacher and friend, and an eccentric scientist whose insights continue to inspire many works in science (Figure 4).

### 3.3 THz active devices and sensing applications

**Jeeyoon Jeong** *Kangwon National University*

**Figure 5:**
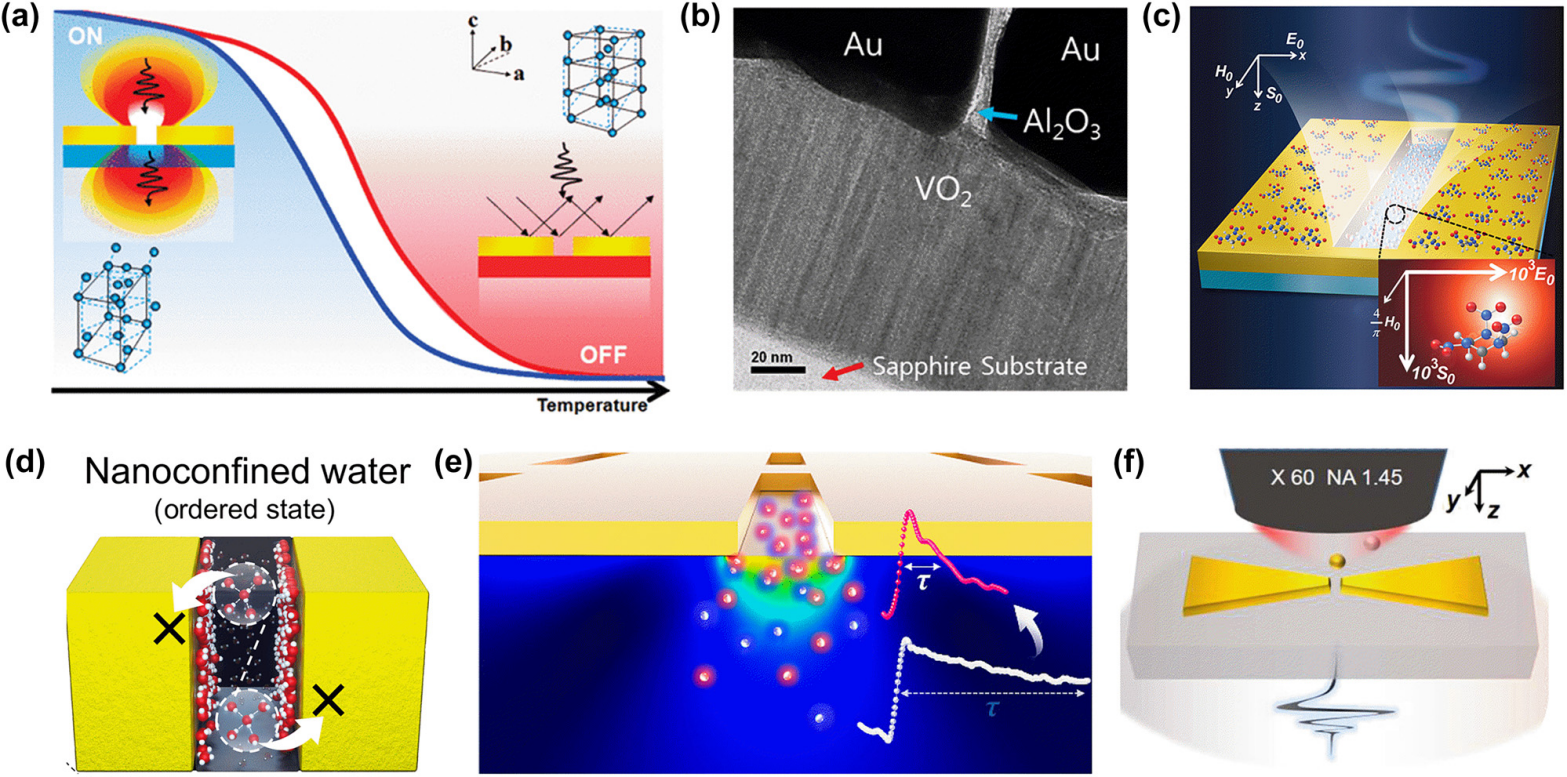
THz plasmonic active metamaterials and high-sensitive sensing. (a) Schematic illustration of the nano slot antennas on a VO_2_ film and its active on/off switching. Reproduced from Ref. [53]. (b) SEM image of 5 nm nanogap structure on a VO_2_ film. Reproduced from Ref. [59]. (c) THz nano-slot-induced molecular absorption enhancement by localized electric field. Reproduced from Ref. [61]. (d) Suppressed THz dynamics of water confined in nano-scale gaps. Reproduced from Ref. [65]. (e) Nanoprobing of surface carrier dynamics by using the nanogap patterning on a bulk semiconductor. Reproduced from Ref. [69]. (f) Experimental concept of optical tweezing THz probing for detection of single gold nano particles trapped in a bowtie gap. Reproduced from Ref. [73].

Dai-Sik’s advancements in the field of THz plasmonics have led to many applications in active devices and sensing. In particular, he has extensively studied the integration of the plasmonic structures with vanadium dioxide (VO_2_), a prototypical material exhibiting metal-to-insulator transition. As nanogaps can enhance absorption and reflection from weakly absorbing or reflecting materials, the VO_2_-integrated nanogaps work as highly efficient, thermally controlled switch for THz radiation [53]. The active modulation is also possible with optical pump [54], [55], [56], electrical stimuli [57], and strong THz pulse [58]. Also, when VO_2_ is integrated with ultra-narrow metallic nanogaps (as narrow as 5 nm), the nanogaps modify the optical response of the metal-to-insulator transition so strongly that the resulting hysteresis curve completely separates from the bulk counterpart [59]. Also, by using VO_2_ itself as the plasmonic building block for slit arrays, he developed a multifunctional, THz-transparent window, which can control near-infrared diffraction orders with temperature [60] (Figure 5a and b).

Dai-Sik also focused on the potential applications of THz nanosensing using metal nanogaps. In his earlier investigations on nanogaps containing metallic nano-barriers, he observed that even a minute perturbation could drastically modify the transmission characteristics [41], [42]. This extreme sensitivity was highly advantageous for nanosensing purposes, and he further demonstrated that such nanogaps are remarkably effective in enhancing molecular absorption. Nanogaps significantly enhance the electric field (*E*) while leaving the magnetic field (*H*) nearly untouched, thereby boosting the electric dipole transition rate (proportional to *E*/*H*) [61]. This unique field distribution has enabled several breakthrough applications, including: highly sensitive detection of trace molecules and single metal nanoparticles [61], [62], and high-contrast probing of nanoscale water confined to thicknesses as thin as 10 nm [63], [64]. Additionally, Dai-Sik’s work has revealed suppressed long-range THz dynamics of water molecules, uncovering molecular behavior previously unseen in DC or infrared measurements [65] (Figure 5c and d).

**Geunchang Choi** *Chung-Ang University*

Prof. Dai-Sik Kim has opened new possibilities for realizing subwavelength-scale active devices through the integration of nanogap and semiconductors in THz photonics. His work demonstrated how strong electromagnetic field confinement can significantly enhance the interaction between THz radiation and semiconductor materials, as well as conducting or insulating nanofilms. These enhanced interactions enable highly efficient nonlinear responses, dynamic switching, and sensitive surface probing, which are essential functionalities for the next generation of THz devices.

His research has extended to explore the nonlinear THz response of semiconductor materials under high field strengths and optical excitation. Building on his earlier demonstrations of THz modulation using VO_2_, he investigated field-induced THz modulation in GaAs with nanometer-scale metallic gaps [66]. These studies led to the development of THz nanoprobing techniques and the realization of highly efficient nonlinear responses in semiconductor platforms.

Notably, he reported a significantly enhanced nonlinear THz response in GaAs induced by nanogap-enhanced field confinement. This resulted in stronger carrier acceleration and an enhanced nonlinear response, both critical for THz switching [67]. In another study, efficient electroluminescence in doped GaAs was achieved with a THz metamaterial structure, where localized THz fields promoted radiative recombination, offering a low-power approach for THz sensor [68].

One of his most ingenious contributions is the implementation of THz nanoprobing method using nanogaps to investigate ultrafast carrier dynamics at semiconductor surfaces. These non-destructive measurements provide insight into direct surface observation of bulk semiconductors [69]. Additionally, he revealed that photoexcited carrier responses in metal nanogap-patterned semiconductors, examined through optical pump–THz probe spectroscopy, originated from the field overlap of two different electromagnetic waves confined in the nanogap. This mechanism paves the way for the design of advanced nanogap-based optical devices that operate under multiwavelength excitation [70] (Figure 5e).

**Geon Lee** *Chung-Ang University*

As demonstrated above, Prof. Kim has made remarkable advancements in THz active devices and sensing applications using various plasmonic structures. Beyond his research on active THz control by integrating materials such as VO_2_ and GaAs with “passive” plasmonic structures, he has also focused on active THz modulation using the metal gap itself. Early research demonstrated that thermal expansion of metals in ultra-narrow slot antennas could modulate THz transmission and resonance without the need for external materials [71].

His work on THz plasmonic applications, combined with diverse structures and fabrication techniques, has opened up new avenues for THz sensing technologies. In this context, mechanical flexibility was introduced by designing nanoslit, nanoslot or bowtie antennas on bendable substrates that could reversibly switch their geometries. In particular, for sensing applications, his THz bowtie antennas with mechanical flexibility enabled dual-frequency operation, facilitating the detection of low-concentration molecules for fingerprinting applications [72]. Most recently, Prof. Kim presented a novel platform for highly sensitive, label-free biochemical sensing by combining optical tweezers with THz nanoscale bowties, enabling precise positioning and real-time detection of single gold nanoparticles [73]. In summary, these advances have transformed THz plasmonics into a versatile tool for active control and highly sensitive sensing applications (Figure 5f).

## 4 Nano-gap technology

From a technical perspective, Dai-Sik has made significant contribution in the areas of nanogap fabrication and deformation techniques. By pushing the limits of both gap width and length to extremes, he has opened the door to THz nanophotonics and THz quantum plasmonics.

### 4.1 Atomic layer lithography (ALL)

**Dukhyung Lee** *Mohammed VI Polytechnic University (UM6P)*

**Sunghwan Kim** *UNIST*

**Figure 6:**
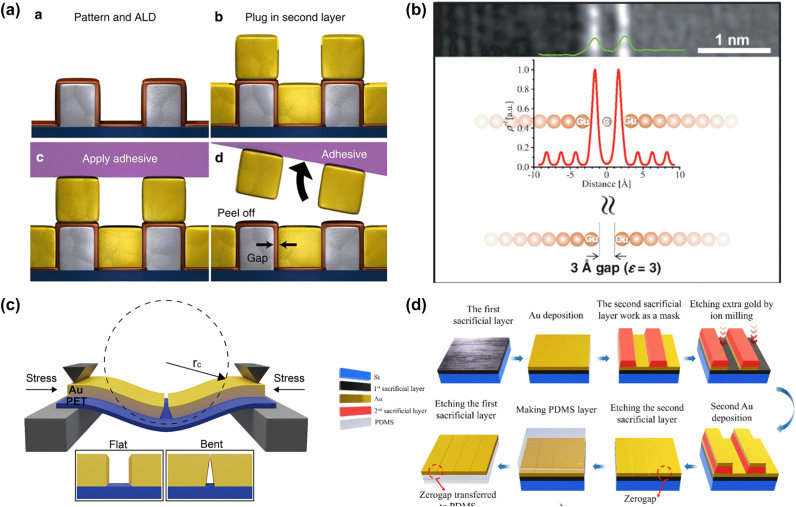
Nano-gap technology. (a) Fabrication steps involved in ALL. Reproduced from Ref. [34]. (b) Angstrom-gap with a CVD-grown graphene spacer. Reproduced from Ref. [37]. (c) Bending of a flexible nano-gap to control its width. Reproduced from Ref. [78]. (d) Zero-gap fabrication and transfer onto a PDMS substrate. Reproduced from Ref. [85].

In the early stages of his pursuit to narrow the gap width, Dai-Sik first employed laser machining to achieve gap widths of a few micrometers [30], [74]. He then turned to focused ion beam (FIB) and electron-beam lithography (EBL) to reach widths down to a few tens of nanometers [25], [39]. At this point, he encountered a technical barrier: conventional lithography techniques struggled to reliably achieve sub-10 nm resolution over typical THz metasurface areas of ∼1 cm^2^. Determined to overcome these limitations, Dai-Sik began desperately exploring new fabrication methods (Figure 6).

In Dai-Sik’s research, the development of atomic layer lithography (ALL) – a powerful technique capable of achieving few-nm gap structures across an entire wafer – enabled the implementation of numerous research studies [34]. The ALL method, which combines standard UV lithography with ALD, is a groundbreaking technique that cost-effectively reduces gap sizes down to sub-10 nm – even as small as 1 nm – overcoming the limitations of conventional nanopatterning techniques such as EBL and FIB at the time. The key idea behind the ALL method is that a very thin aluminum oxide (Al_2_O_3_) insulating layer is conformally deposited along the pattern surface through a self-limiting reaction. As a result, the nano-gap size can be precisely controlled on nm-scale by adjusting the thickness of the insulating layer, which is determined by the number of deposition cycles in the ALD process. Since the first report in 2013, the ALL method has undergone continuous improvements, introducing techniques such as ion milling and chemical wet etching to expand its range of applications involving various metal species and pattern designs. More recently, the direct use of photoresist as a mask has also been introduced [75], [76], [77]. Furthermore, the ALL method was adapted to achieve an angstrom-scale gap using a graphene sheet as a gap material, by replacing the ALD with chemical vapor deposition (CVD) [37], [38]. Interestingly, we found that the gap width could be tuned on the angstrom scale by controlling the number of graphene layers due to van der Waals distance between them (Figure 6a and 6b).

### 4.2 Flexible metal gap

**Dukhyung Lee** *UM6P*

After developing ALL, Dai-Sik started to deliberate how the nano-gaps can be actively controlled, which could vastly expand the application range. Dai-Sik developed various active control schemes, including optical, electrical and thermal methods. Among them, the one he had devoted to the last moment was mechanical deformation. In 2020, Dai-Sik and his colleagues applied ALL on PET substrates to fabricate closable nano-gaps that can be adjusted by mechanical bending of the substrate [78]. Here, the alumina spacers were etched out to enable direct contact between the side metals. Conductance between the two metal sides increased upon inward bending of the PET substrate, while transmission through the nano-gap decreased across a broadband spectrum, achieving zero transmission in the THz and microwave regimes. By embedding closable nanogaps into THz metasurfaces, it became possible to switch their optical responses, such as resonance frequency or polarization dependence. Following the demonstration of flexible nano-gaps on PET substrates, Dai-Sik expanded his scope to PDMS substrates, which offer the advantages of being both bendable and stretchable [79], [80] (Figure 6c).

It is worth pointing out the distinction between breakjunctions formed by rupturing and flexible nanogaps fabricated by ALL. While breakjunctions can be used in a similar manner to impart switchability to metasurfaces [72], what Dai-Sik noticed and was excited about is that, due to the inherent roughness of the metal sidewalls, mm-long nano-gap lines can operate as an extremely large number of quantum point contacts, all controllable at once. An immediate application of this feature was THz quantum plasmonics on an mm-scale, and it still holds potential for further discoveries and implementations in the emerging quantum era.

### 4.3 Zero-gap approaches

**Jeeyoon Jeong** *Kangwon National University*

Dai-Sik’s journey to make the narrowest gap possible has finally led to a concept of ‘zero-gap’ – that is, to start from a bare metallic film and crack open the gap by using a flexible substrate. As the idle state of a zero-gap is ‘off’ state, zero-gaps generally show much better contrast compared to ordinary flexible gaps [81]. Also, fabrication is simpler as zero-gaps do not require ALD process; deposition of two metallic layers at different times create a subtle boundary between the two layers, which separates upon gently bending the substrate [82]. Such zero-gaps have been utilized in advanced surface-enhanced Raman spectroscopy [83], [84]. Also, they are found to be useful as strain sensors [85] (Figure 6d).

## 5 A mentor who shaped researchers

**Young-Gyun Jeong** *University of Toronto*

There is a common saying in sports that star players rarely make great coaches. But Professor Dai-Sik Kim was a rare exception – an outstanding researcher in his own right and a deeply dedicated mentor. Many of his former students, myself included, have continued along the path of research, a testament to both the example he set and the thoughtful guidance he provided. He not only embodied what it meant to be a good researcher but also instilled in his students the mindset and habits essential for a meaningful scientific career.

I was far from an exceptional graduate student, and I still have many shortcomings as a researcher. Yet, I am certain that I would not have remained in this profession had I not been mentored by Prof. Kim. His influence was that profound. In the reflections that follows, I would like to briefly share the philosophy that shaped his approach to mentoring, the mindset he sought to instill in his students, and the aspirations he pursued as a mentor – understood through the lens of my own experience.

### 5.1 You learn by failing

When I was a graduate student, my fear of failure often held me back from making genuine attempts. I had been accustomed to solving well-defined problems and receiving positive feedback for correct answers. Research, however, offered none of that clarity or reassurance. Confronted with tasks that seemed destined to fail or beyond my understanding, I would often freeze – choosing inaction over the risk of failure. This habit, born of fear, kept me confined to the safe area of what I already knew. In retrospect, this was a serious obstacle to becoming a researcher.

My mentor never stopped trying to change this in me. He consistently challenged my passivity and fostered in me a mindset of ownership and determination. When I would say, “I’ll try,” he would urge me to replace it with, “I will do it.” It took years for me to fully understand that in research, failure is not something to fear – it is the very mechanism by which we learn and progress. Without his persistent encouragement and discipline, I might never have embraced that lesson.

### 5.2 Forward thinking

Early in my academic journey, I had a limited understanding of what it meant to be a teacher – or a mentor. I believed that a teacher’s role was to instruct, encourage, and provide the right answers. Entering graduate school with this mindset, I assumed my advisor already knew the solutions to the problems I was working on, and that my task was merely to find and confirm those answers. In hindsight, this was a narrow and passive way of thinking.

Because of this mindset, I rarely engaged deeply with the research problems I faced. I did not take the initiative to explore new directions to question underlying assumptions. Instead, I focused on following instructions and completing tasks as assigned. But research seldom advances so neatly. Experimental results often raise new questions, demand interpretation, and require charting an uncertain path forward. Yet, when things did not go as planned, my default response was simply, “I tried it, but it didn’t work.” Even by the end of my Ph.D., I cannot recall ever proposing a new approach or idea before my mentor did.

It was only after leaving his lab and beginning my postdoctoral work that I truly grasped how much initiative and independent thinking research demands. In retrospect, I can see how much effort he invested in helping me develop this forward-thinking mindset. He never stopped urging me to take ownership of my work and to think beyond the immediate task at hand. Through his patience and unwavering sense of responsibility as a mentor, I gradually came to understand – though belatedly – what it means to be proactive, to anticipate challenges, to lead one’s own research, and to approach science with both intention and creativity.

### 5.3 You must see it through to the end

Even in research, most people are guided by an instinct for self-preservation. It is human nature to prefer comfort over discomfort, and in the scientific world – where failure is frequent – there is a strong temptation to stop once a result seems “good enough.” When outcomes fall short of expectations, it is equally easy to rationalize, blaming external factors or circumstances, and to feel content simply because an effort was made.

Prof. Kim consistently challenged this tendency. He taught that research demands not only unwavering integrity, but also the courage to break past one’s own perceived limits – to go further than what feels safe or comfortable. He urged us to keep questioning our assumptions, to test whether our conclusions truly held, and to push our work beyond the point where most would stop. His goal was that we could stand by our results with complete conviction, knowing they had been pursued to their fullest possible extent. This often meant stepping outside conventional, low-risk choices and embracing uncertainty, complexity, and even discomfort, all in pursuit of the clearest possible truth.

This philosophy was not abstract – it shaped countless decisions at the lab bench and in the publication process. I vividly recall one instance during the review of our 2015 Nano Letters paper [59], which investigated changes in the phase transition temperature of nanogap-patterned VO_2_. A reviewer asked whether the observed shift in transition temperature was truly due to the nanogap itself, or instead from intrinsic changes in the VO_2_ caused by the nanogap fabrication process. Rather than opting for a workaround or offering an indirect explanation – the easier and more common approach – he instructed me to etch away the nanogap and remeasure the phase transition temperature. Sacrificing a painstakingly fabricated sample is something most researchers would avoid, but for him, certainty outweighed preservation. What others might hesitate to do, I came to see as the embodiment of his research philosophy: to take the most straightforward and truthful path, and to push through whatever internal barriers stood in the way, regardless of the risk of loss or the extra effort required. For him certainty was possible only through integrity and the courage to go beyond.

### 5.4 On the independence and self-reliance of scholarship

Prof. Kim’s mentoring style, as I experienced it, resembled the way an eagle teaches its young to fly. Rather than providing a friendly step-by-step guide, he encouraged us to learn through trial and error in the real world. For me, it often felt as though there were more sticks than carrots. It reminded me of the traditional image of a stern Korean father – demanding, principled, and exacting.

Over time, however, it became clear how deeply he cared for his students. Beneath his firm exterior, he supported us quietly and steadily – acknowledging our personal milestones, guiding us through difficult decisions, and helping alumni navigate their careers long after graduation. Hearing about these small but meaningful acts – often secondhand – revealed a deeply human and compassionate side of him that was not always visible on the surface.

Prof. Kim’s ultimate goal as a mentor was to nurture truly independent researchers. He often voiced concerns about Korea’s educational landscape, especially the tendency for many of the country’s brightest students to pursue degrees abroad, return to Korea as professors, yet continue to rely on the networks and systems they had built overseas. He was critical of this dependency. What he envisioned instead was a Korea capable of nurturing its own world-class scholars – researchers shaped and supported by domestic institutions, thriving within a self-sustaining research ecosystem.

With this vision, he sought to create in his own lab an environment that met global standards. He provided not only intellectual rigor but also the professional conditions that allowed students to grow into independent thinkers. Many of his former students have since gone on to build successful careers as researchers. While I never heard him express overt patriotic sentiments, I believe his mentorship philosophy – and the deeply human values behind – were, in their own quiet way, a powerful expression of his identity as a Korean and his love for his country.

As someone who benefited immensely from his passionate and principled mentorship, I offer my deepest respect and gratitude. I mourn his passing with profound sorrow, and yet I also hope that in heaven he finds joy in seeing his legacy and vision carried forward and flourishing. May you rest in peace, my mentor.

## 6 Final thoughts

**Young-Mi Bahk** *Incheon National University*

In addition to Professor Dai-Sik Kim’s academic achievements in the field of nano-optics discussed earlier, I would like to conclude this article by reflecting on key moments from his journey as a scientist, teacher, and social activist.

First, he was a true pioneer. Leaving behind a well-established reputation in femtosecond dynamics (quantum mechanics), he made the bold decision to shift his research focus to a completely different field – nanooptics and plasmonics (electromagnetism). This transition demanded exceptional courage and vision. His move from Seoul National University, belonging to the top class universities in Korea, to UNIST, founded newly in southern Korean peninsula, was also a symbolic step. At the time, this decision was unprecedented in Korean society and sent a powerful message about his commitment to innovation and challenge. Driven by this spirit of challenge, he left the honors of a traditional physicist behind in the latter part of his career and turned toward applied research, exploring new possibilities.

**Figure 7:**
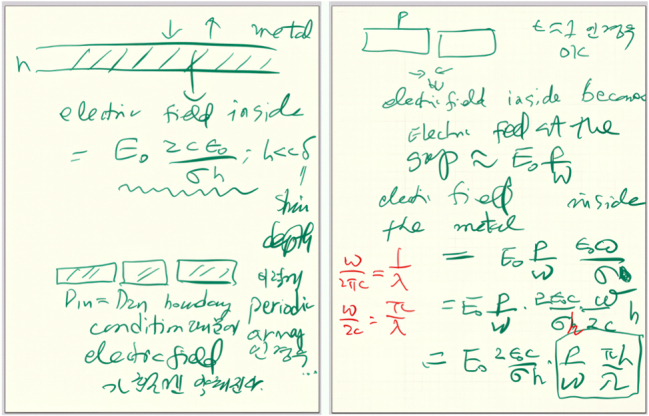
Dai-Sik Kim’s handwritten notes on the behavior of electric fields in metal-dielectric structures.

Second, he was a technological innovator. His research pushed the boundaries of optics beyond the diffraction limit, an achievement made possible by continuous technological advancements. With breakthroughs in fabrication techniques, creative ideas, and a relentless drive to challenge conventional wisdom, he crafted a research narrative spanning micrometers, nanometers, angstroms, and even zero-gap. One of his most memorable insights was that “Physicists should have greater expertise in technology than engineers.” This reflects his scientific philosophy of simultaneously pursuing both theoretical depth and technological innovation (Figure 7).

Lastly, he was also a social activist. He raised public awareness about circumcision in Korean society and courageously criticized deep-rooted issues within Korea’s educational system. Through these efforts, he challenged longstanding societal norms and vested interests, offering fresh and often uncomfortable perspectives. While his individual actions may not have brought about immediate, large-scale change, the new viewpoints and issues he raised as one of Korea’s leading scientists left a lasting impact. His insights remain an invaluable intangible legacy for future generations.

Finally, as one of Prof. Dai-Sik Kim’s students, it was a great honor to contribute, however modestly, to his research journey. I deeply mourn his passing.
